# Risk of renal cell carcinoma in relation to blood telomere length in a population-based case–control study

**DOI:** 10.1038/bjc.2011.444

**Published:** 2011-10-27

**Authors:** J N Hofmann, A Baccarelli, K Schwartz, F G Davis, J J Ruterbusch, M Hoxha, B J McCarthy, S A Savage, S Wacholder, N Rothman, B I Graubard, J S Colt, W-H Chow, M P Purdue

**Affiliations:** 1 Division of Cancer Epidemiology and Genetics, Occupational and Environmental Epidemiology Branch, National Cancer Institute, 6120 Executive Boulevard, EPS 8109, Bethesda, MD 20892-7240, USA; 2 Department of Environmental Health, Harvard School of Public Health, Boston, MA, USA; 3 Department of Family Medicine and Public Health Sciences, Karmanos Cancer Institute, Wayne State University, Detroit, MI, USA; 4 Division of Epidemiology and Biostatistics, School of Public Health, University of Illinois at Chicago, Chicago, IL, USA; 5 Department of Occupational and Environmental Health, University of Milan and Ca’ Granda Ospedale Maggiore Policlinico Hospital IRCCS Foundation, Milan, Italy

**Keywords:** telomere, renal cell carcinoma, kidney cancer

## Abstract

**Background:**

There are few known risk factors for renal cell carcinoma (RCC). Two small hospital-based case–control studies suggested an association between short blood telomere length (TL) and increased RCC risk.

**Methods:**

We conducted a large population-based case–control study in two metropolitan regions of the United States comparing relative TL in DNA derived from peripheral blood samples from 891 RCC cases and 894 controls. Odds ratios and 95% confidence intervals were estimated using unconditional logistic regression in both unadjusted and adjusted models.

**Results:**

Median TL was 0.85 for both cases and controls (*P*=0.40), and no differences in RCC risk by quartiles of TL were observed. Results of analyses stratified by age, sex, race, tumour stage, and time from RCC diagnosis to blood collection were similarly null. In multivariate analyses among controls, increasing age and history of hypertension were associated with shorter TL (*P*<0.001 and *P*=0.07, respectively), and African Americans had longer TL than Caucasians (*P*<0.001).

**Conclusion:**

These data do not support the hypothesis that blood TL is associated with RCC. This population-based case–control study is, to our knowledge, the largest investigation to date of TL and RCC.

Telomeres are nucleotide repeats and a protein complex at chromosome ends that are essential for chromosomal stability. Telomere attrition occurs with each cell division due to inefficient replication at the ends of linear DNA. Critically short telomeres trigger cellular senescence and death, but cancer cells divide despite the resultant genomic instability. Telomere length (TL) is a suspected marker of cancer risk (De Lange, 2005). Two small hospital-based case–control studies (32 and 65 cases, respectively) reported an increased risk of renal cell carcinoma (RCC) in relation to shorter TL (Wu *et al*, 2003; Shao *et al*, 2007). To follow up on these findings, we evaluated RCC risk in relation to TL in a large case–control study.

## Materials and methods

### Study population, data and sample collection, and sample processing

Subject recruitment and data and specimen collection methods have been described (Colt *et al*, 2011). Briefly, this population-based case–control study of Caucasians and African Americans was conducted in Detroit, MI, USA (Wayne, Oakland, and Macomb Counties) from 2002–2007, and in Chicago, IL, USA (Cook County) in 2003; according to US census estimates from 2000, the populations of these two metropolitan areas had generally similar racial distributions (56.3% Caucasian and 26.1% African American in Cook County; 68.9% Caucasian and 25.0% African American in Wayne, Oakland, and Macomb Counties, combined). To maximise enrolment of African Americans, we over-sampled African American cases relative to Caucasian cases, and we frequency matched controls to cases at a 2 : 1 ratio for African Americans and a 1 : 1 ratio for Caucasians to increase statistical power for analyses stratified by race. Subjects with stored blood samples (whole blood or buffy coat) were included in this analysis. Eight cases with benign tumours, non-RCC histology, or cancer in a transplanted kidney were excluded. Telomere length could not be measured for one control subject, who was also excluded, leaving 891 cases (658 Caucasians and 233 African Americans) and 894 controls (550 Caucasians and 344 African Americans). Blood samples were collected from cases and controls at the time of the personal interview. Among cases, the median time from RCC diagnosis to blood sample collection was ∼4 months. Samples of DNA were extracted via Qiagen kits; DNA was derived from whole blood samples for most study subjects (cases: 627 whole blood, 264 buffy coat; controls: 768 whole blood, 126 buffy coat). The distribution of the source material for DNA extraction was similar for each study centre. Study procedures were approved by Institutional Review Boards at collaborating institutions, and written informed consent was obtained from all subjects.

### TL measurements

A quantitative PCR assay was used to measure TL; assay methods have been described (Cawthon, 2002). Briefly, telomere repeat (T) and single gene (S) copy numbers were measured in individual samples and adjusted in comparison to standard reference DNA; the standardised T/S ratio characterises relative TL. In TL measurements for blinded duplicate QC samples from 59 subjects, the coefficient of variation (CV) was 9.9% and the intraclass correlation coefficient was 0.85 (95% confidence interval (CI): 0.76, 0.91).

### Statistical analysis

Telomere length data were natural log-transformed to achieve a normal distribution. We compared TL between cases and controls, and evaluated differences in TL by demographic and personal characteristics among controls in bivariate and multivariate analyses. Odds ratios (ORs) and 95% CIs were calculated using unconditional logistic regression. Quartiles of TL were determined based on the distribution among controls. Adjusted analyses included terms for age (10-year categories), sex, race, smoking, body mass index (BMI), history of hypertension, education, study centre, and material type (whole blood or buffy coat). Analyses stratified by these covariates, tumour stage/grade, RCC treatment modality, and time from RCC diagnosis to blood collection were also performed.

## Results

Cases and controls had similar age and sex distributions. Cases were more likely to be obese (BMI⩾30), to smoke, and to have a history of hypertension, as reported previously (Karami *et al*, 2010). The vast majority of cases with treatment information available had surgery alone without adjuvant therapy (*N*=803; 92%), and most cases with information on stage at diagnosis had localised disease (*N*=611; 81%).

Median relative TL (5th–95th percentile distributions) was 0.85 (0.58–1.25) and 0.85 (0.58–1.23) for cases and controls, respectively (*P*=0.40, Wilcoxon rank sum test). A box plot showing the distribution of TL measurements among cases and controls is available online (Supplementary Figure 1). No differences in TL between cases and controls were observed after stratifying by material type. The expected age-related decline in TL was observed in both cases and controls. In multivariate analyses among controls, TL was significantly longer among African Americans than among Caucasians (*P*<0.001), and we observed a borderline significant association between hypertension and shorter TL (*P*=0.07; Table 1).

No overall associations between TL and RCC were observed (Table 2). Analyses stratified by sex, race, age, or other variables did not reveal any consistent subgroup-specific associations between TL and RCC. No differences in the relationship between TL and RCC by tumour stage (localised *vs* other) were observed, nor when we restricted our analysis to cases treated by surgery alone. We did not observe any differences in TL by days from RCC diagnosis to blood collection (adjusted *β*=−2.95 × 10^−5^; 95% CI: −9.01 × 10^−5^, 3.11 × 10^−5^), and risk estimates did not differ after stratifying by time since diagnosis (data not shown).

## Discussion

The results of this case–control study do not support the hypothesis that blood TL is associated with RCC. Our study did not replicate findings from two hospital-based case–control studies with 32 and 65 RCC cases, respectively, that reported an inverse association between TL and RCC (Wu *et al*, 2003; Shao *et al*, 2007). Both prior studies measured TL using a Q-FISH assay; it has been demonstrated that Q-FISH measurements are highly correlated with measurements by the QPCR method used in this study (Cawthon, 2002). Because measurements in our study were highly reproducible in blind replicates (CV=9.9%), it is unlikely that measurement error could explain the difference in findings between our study and previous studies.

This population-based case–control study is, to our knowledge, the largest investigation of TL and RCC to date. We had 89% power to detect a trend in ORs with decreasing quartiles of TL assuming an OR of 1.5 comparing the lowest and highest quartiles. Our findings of shorter TL with increasing age and history of hypertension are consistent with previous reports (Demissie *et al*, 2006; Mirabello *et al*, 2009), which supports the validity of these findings. Differences in TL by race are inconsistent in previous studies (Hunt *et al*, 2008; Roux *et al*, 2009) and additional research is needed to confirm these findings. Numerous studies have investigated TL in relation to smoking and BMI (Wu *et al*, 2003; Valdes *et al*, 2005; Nordfjall *et al*, 2008; Kim *et al*, 2009; Mirabello *et al*, 2009; Fitzpatrick *et al*, 2011; Lee *et al*, 2011; Shen *et al*, 2011). Overall, the totality of the evidence linking these exposures to TL is inconsistent, with only some studies reporting inverse associations with smoking (Valdes *et al*, 2005; Mirabello *et al*, 2009; Fitzpatrick *et al*, 2011; Shen *et al*, 2011) and BMI (Valdes *et al*, 2005; Nordfjall *et al*, 2008; Kim *et al*, 2009; Lee *et al*, 2011).

Measurement of TL in samples collected retrospectively is an inherent limitation of case–control studies. Previous studies of various cancers have reported strong associations between short TL and cancer risk in retrospective studies but not in studies with prospective sample collection (Pooley *et al*, 2010; Wentzensen *et al*, 2011). However, in our study, we did not observe any differences in the relation between TL and RCC after stratifying by tumour stage, tumour grade, and time from RCC diagnosis to blood collection, nor when we restricted to cases treated by surgery only. Furthermore, Svenson *et al* (2009) found that among cases with non-metastatic disease (consistent with most of the cases included in our study) TL was not related to survival until >10 months after RCC diagnosis. Since the vast majority of samples in our study were collected from RCC cases within 10 months of diagnosis, we would not expect our findings to be biased as a result of differential survival. Moreover, since long TL was associated with poor survival, any bias due to a survival effect would be expected to exaggerate (rather than to obscure) an association between short TL and RCC risk. Given this evidence from our study and the analysis by Svenson *et al* (2009), it is unlikely that disease- or treatment-related changes in TL would have affected our findings. In conclusion, we found no evidence of an association between blood TL and RCC risk in this population-based case–control study, to our knowledge the largest such investigation to date.

## Figures and Tables

**Table 1:** Determinants of blood telomere length among controls^a^

		**Bivariate analysis**	**Multivariate analysis^b^**
**Variable**	***N***	**Geometric mean (95% CI)**	**Difference in geometric mean (95% CI)^c^**
*Age category*			
<45	128	1.00 (0.97, 1.04)	Ref
45–54	198	0.88 (0.85, 0.91)	–10% (–15%, –6%)^*^
55–64	255	0.82 (0.80, 0.85)	–15% (–19%, –11%)^*^
65–74	237	0.79 (0.77, 0.82)	–18% (–22%, –14%)^*^
75+	76	0.78 (0.74, 0.83)	–18% (–23%, –13%)^*^
		*P*_trend_<0.001	*P*_trend_<0.001
			
*Sex*			
Female	381	0.86 (0.84, 0.89)	Ref
Male	513	0.83 (0.82, 0.85)	–2% (–5%, 1%)
			
*Race*			
Caucasian	550	0.82 (0.81, 0.84)	Ref
African American	344	0.89 (0.86, 0.91)	9% (5%, 12%)^*^
			
*Hypertension*			
Never	525	0.87 (0.85, 0.89)	Ref
Ever	364	0.81 (0.79, 0.83)	–3% (–6%, 0%)^**^
			
*Smoking status*			
Never	346	0.86 (0.84, 0.89)	Ref
Occasional	41	0.87 (0.80, 0.95)	–1% (–7%, 6%)
Former	331	0.83 (0.81, 0.85)	0% (–3%, 3%)
Current	175	0.85 (0.82, 0.88)	–2% (–6%, 2%)
			
*BMI*			
<25	256	0.85 (0.83, 0.88)	Ref
25–29.9	366	0.84 (0.82, 0.86)	2% (–2%, 5%)
30–34.9	155	0.86 (0.82, 0.89)	3% (–2%, 7%)
35+	114	0.84 (0.80, 0.88)	0% (–5%, 5%)
			
*Source of DNA specimen*			
Whole blood	768	0.83 (0.82, 0.85)	Ref
Buffy coat	126	0.95 (0.91, 0.99)	10% (5%, 15%)^*^

Abbreviations: CI=confidence interval; BMI=body mass index.

a Telomere length measurements were expressed as the standardised T/S ratio, and data were natural log-transformed for all analyses.

b Each variable was evaluated after adjusting for all other covariates reported above as well as study centre and level of education. Nine controls with missing data for any variable were excluded from this analysis.

c The percent difference in the geometric mean relative to the reference category was estimated using the formula (exp(*β*)–1).

^*^P<0.001.

^**^P=0.07.

**Table 2:** Risk of renal cell carcinoma in relation to blood telomere length^a^

**Telomere length quartile^b^**	***N*_cases_**	***N*_controls_**	**Unadjusted OR (95% CI)**	**Adjusted OR (95% CI)^c^**
*Overall*				
4th Quartile	259	222	Ref	Ref
3rd Quartile	177	224	0.68 (0.52, 0.88)	0.69 (0.51, 0.93)
2nd Quartile	242	224	0.93 (0.72, 1.20)	0.90 (0.67, 1.20)
1st Quartile	213	224	0.82 (0.63, 1.06)	0.79 (0.59, 1.07)
			*P*_trend_=0.29	*P*_trend_=0.25
				
*Stratified analyses*				
Sex				
Women				
4th Quartile	116	102	Ref	Ref
3rd Quartile	80	108	0.65 (0.44, 0.96)	0.68 (0.43, 1.09)
2nd Quartile	104	82	1.12 (0.75, 1.65)	1.11 (0.69, 1.79)
1st Quartile	69	89	0.68 (0.45, 1.03)	0.60 (0.36, 0.99)
			*P*_trend_=0.28	*P*_trend_=0.17
				
Men				
4th Quartile	143	120	Ref	Ref
3rd Quartile	97	116	0.70 (0.49, 1.01)	0.73 (0.49, 1.08)
2nd Quartile	138	142	0.82 (0.58, 1.14)	0.82 (0.56, 1.19)
1st Quartile	144	135	0.90 (0.64, 1.25)	0.90 (0.61, 1.32)
			*P*_trend_=0.59	*P*_trend_=0.66
				
Race				
African American				
4th Quartile	80	107	Ref	Ref
3rd Quartile	44	86	0.68 (0.43, 1.09)	0.73 (0.43, 1.26)
2nd Quartile	57	87	0.88 (0.56, 1.36)	0.73 (0.43, 1.23)
1st Quartile	52	64	1.09 (0.68, 1.73)	0.84 (0.48, 1.46)
			*P*_trend_=0.85	*P*_trend_=0.42
				
Caucasian				
4th Quartile	179	115	Ref	Ref
3rd Quartile	133	138	0.62 (0.44, 0.86)	0.69 (0.48, 0.99)
2nd Quartile	185	137	0.87 (0.63, 1.20)	0.97 (0.68, 1.39)
1st Quartile	161	160	0.65 (0.47, 0.89)	0.76 (0.53, 1.10)
			*P*_trend_=0.04	*P*_trend_=0.32
				
Age				
Under 60 years of age				
4th Quartile	177	160	Ref	Ref
3rd Quartile	99	123	0.73 (0.52, 1.02)	0.81 (0.55, 1.20)
2nd Quartile	103	105	0.89 (0.63, 1.25)	1.00 (0.67, 1.49)
1st Quartile	80	67	1.08 (0.73, 1.59)	1.29 (0.83, 2.02)
			*P*_trend_>0.99	*P*_trend_=0.36
				
60+ years of age				
4th Quartile	82	62	Ref	Ref
3rd Quartile	78	101	0.58 (0.38, 0.91)	0.60 (0.38, 0.96)
2nd Quartile	139	119	0.88 (0.59, 1.33)	0.85 (0.55, 1.32)
1st Quartile	133	157	0.64 (0.43, 0.96)	0.62 (0.40, 0.95)
			*P*_trend_=0.13	*P*_trend_=0.09
				
Source of DNA specimen				
Whole blood^d^				
4th Quartile	145	192	Ref	Ref
3rd Quartile	139	192	0.96 (0.71, 1.30)	0.78 (0.55, 1.10)
2nd Quartile	180	192	1.24 (0.92, 1.67)	0.88 (0.63, 1.23)
1st Quartile	163	192	1.12 (0.83, 1.52)	0.79 (0.56, 1.11)
			*P*_trend_=0.23	*P*_trend_=0.25
				
Buffy coat^e^				
4th Quartile	72	32	Ref	Ref
3rd Quartile	55	31	0.79 (0.43, 1.45)	0.68 (0.35, 1.32)
2nd Quartile	68	32	0.94 (0.52, 1.71)	0.80 (0.41, 1.57)
1st Quartile	69	31	0.99 (0.55, 1.79)	1.11 (0.56, 2.21)
			*P*_trend_=0.90	*P*_trend_=0.70

Abbreviations: OR=odds ratio; CI=confidence interval; BMI=body mass index.

a Telomere length measurements were expressed as the standardised T/S ratio.

b Quartiles were determined based on the distribution of telomere length measurements among controls. Cut points were defined as follows: Q1, ⩽0.7288; Q2, 0.7289–0.8535; Q3, 0.8536–0.9795; and Q4, ⩾0.9796.

c Adjusted for the following covariates: sex, age, race, smoking status, BMI, history of hypertension, level of education, study centre (Detroit or Chicago), and material type (whole blood or buffy coat). In all, 30 subjects with missing data for smoking status, BMI, or history of hypertension were excluded from this analysis.

d Quartiles based on the distribution of telomere length measurements among controls with whole blood samples. Cut points were defined as follows: Q1, ⩽0.7197; Q2, 0.7198–0.8341; Q3, 0.8342–0.9633; and Q4, ⩾0.9634.

e Quartiles based on the distribution of telomere length measurements among controls with buffy coat samples. Cut points were defined as follows: Q1, ⩽0.8323; Q2, 0.8324–0.9651; Q3, 0.9652–1.1048; and Q4, ⩾1.1049.
