# Supplementary material for: Risk of renal cell carcinoma in relation to blood telomere length in a population-based case–control study
Source: Br J Cancer. 2011 Oct 27;105(11):1772–5. doi: 10.1038/bjc.2011.444 (PMC3242602; doi:10.1038/bjc.2011.444)
Supplement: Supplementary Figure 1 [file bjc2011444x1.doc]

**Supplemental Figure 1. Box plot of relative telomere length by case-control status**
